# Measuring quality of life after intensive care using the Arabic version for Morocco of the EuroQol 5 Dimensions

**DOI:** 10.1186/1756-0500-5-56

**Published:** 2012-01-22

**Authors:** Ibtissam Khoudri, Jihane Belayachi, Tarek Dendane, Khalid Abidi, Naoufel Madani, Aicha Zekraoui, Amine Ali Zeggwagh, Redouane Abouqal

**Affiliations:** 1 Faculty of Medicine, Laboratory of Biostatistics Clinical and Epidemiological Research, Rabat, Morocco; 2 Medical Emergency Department, Ibn Sina University Hospital, Rabat, Morocco; 3 Medical Intensive Care Unit, Ibn Sina University Hospital, Rabat, Morocco

**Keywords:** EQ-5D, Intensive care, Quality of life

## Abstract

**Background:**

Health-related quality of life (HRQL) is a relevant outcome measures in intensive care unit (ICU). The aim of this study was to evaluate HRQL of ICU patients 3 months after discharge using the Arabic version for Morocco of the EuroQol-5-Dimension (EQ-5D), and to examine the psychometric properties of the questionnaire.

**Results:**

The Arabic version for Morocco of the EQ-5D was approved by the EuroQol group. A prospective cohort study was conducted after medical ICU discharge. At 3-month follow up, the EQ-5D (self classifier and EQ-VAS) was administered in consultation or by telephone. EQ-VAS varies from 0 (better HRQL) to 100 (worst HRQL). An unweighted scoring for EQ5D-index was calculated. EQ5D-index ranges from -0.59 to 1. Test-retest reliability of the EQ-5D was tested using Kappa coefficient and intraclass correlation coefficient (ICC). Criterion validity was assessed by correlating EQ-VAS and EQ5D-index with the Short Form 36 (SF-36). Construct validity was tested using simple and multiple liner regression to assess factors influencing patients'HRQL. 145 survivors answered the EQ-5D. Median EQ5D-index was 0.52 [0.20-1]. Mean EQ-VAS was 62 ± 20. Test-retest reliability was conducted in 83 patients. ICCs of EQ5D-index and EQ-VAS were 0.95 and 0.92 respectively. For EQ-5D self classifier, agreement by kappa was above 0.40. Significant correlations were noted between EQ5D-index, EQ-VAS and SF-36 (*p *< 0.001). In multivariate analysis, factors associated with poorer HRQL for EQ5D-index were longer ICU length of stay (β = -0.01; *p *= 0.017) and higher educational level (β = -0.2; *p *= 0.001). For EQ-VAS men were associated with better HRQL (β = 6.5; *p *= 0.048).

**Conclusions:**

The Arabic version for Morocco of the EQ-5D is reliable and valid. Women, high educational level and longer ICU length of stay were associated with poorer HRQL.

## Background

Assessing outcome after intensive care unit (ICU) care is notoriously difficult [1]. Objective study end points such as mortality are relatively easy to assess. Nevertheless, these may not be the only outcomes of interest to patients, families, and healthcare providers. If ICU treatment leads to deterioration in the quality of life, patients may be concerned that small gains in life expectancy come at too high a cost. Other outcome measures such as health-related quality of life (HRQL) are then imperative to medical evaluation [2]. HRQL is actually one of the most relevant outcome measures for patients, families, physicians and society [3].

Several scales have been used to measure the different domains of HRQL; some are generic while others are disease-specific. The generic scales attempt to capture aspects of health that are important to all patients and therefore present the advantage of comparing different groups of patients [1-4]. The EuroQol -5 Dimensions (EQ-5D) is a generic questionnaire that has been developed by an international task force aiming at creating a simple generic measure that aggregated HRQoL into a single index [5]. The EQ-5D has been already used in numerous studies and in subsets of critically ill patients [3]. The validity and reliability of the EQ have been tested in the ICU population, and it has been recommended for use in critical care medicine [6-10].

The EQ-5D is available in many major languages with cultural adaptations [11-14]. Although HRQL data are widely available on a number of western countries, few data are available in Arabic countries [15]. In North-African and Arabic countries, few HRQL studies have been published in ICUs, a Moroccan study have focused on measuring HRQL after intensive care using the Short-Form 36 (SF-36) [16]. However, to our knowledge this is the first validation study of the EQ-5D in North-African and Arabic ICUs.

This study aimed to evaluate the HRQL of a Moroccan cohort' patients alive 3 months after ICU discharge; to study its determinants and to assess the psychometric properties of the Arabic version for Morocco of the EQ-5D.

## Methods

### Study design, setting and subjects

This was a prospective study of Adult patients who were discharged between November 2004 and August 2005 from a medical ICU of Rabat University Hospital. The 12-bed medical ICU admits approximately 550 patients annually with an average age of 40 years. Surgery patients, coronary, neonates and burn patients are treated in specialized units. Rabat University Hospital is referral for habitants in Western-North Morocco. The protocol was approved by the Hospital Ethics Committee, and informed consent was obtained from all participants.

Inclusion criteria were adult patients (> 18 years old) with an ICU stay of more than 24 h. Patients who died during ICU stay were excluded. Patients discharged alive from ICU were asked to attend a consultation 3 months later. Survivors were then administered the EQ-5D in consultation at the 3-month follow up. If patients did not come to the follow-up consultation, a telephone contact was established for a follow-up interview. We have chosen to assess HRQL at 3 months because it seems to be ideal in the detection of only ICU-related morbidity and permits early psychological intervention if required [1]. In consultation, questionnaires were self-completed by the patients with a high education level (secondary/higher) or administrated by the same investigator if the education level is lower (none/primary). No proxy assessment was allowed. Follow-up interviews were always conducted by the same interviewer in order to ensure the EQ-5D reliability and minimize missing values.

### Data collection

Background variables at admission included patient demographic data and pre-admission comorbidity classified using the Charlson Comorbidity Index (CCI) [17]: among severely ill patients, a CCI score of two or greater is associated with 1-year survival of less than 20%. Primary diagnosis, severity of disease assessed by the Acute Physiology and Chronic Health Evaluation II (APACHE II) [18], Therapeutic Intervention Scoring System (TISS) scores [19], use of mechanical ventilation and length of stay in the ICU were also recorded.

### EQ-5D, translation and cultural adaptation process

The first part of the EQ-5D called "self-classifier" covers five dimensions, namely mobility, self-care, usual activities, pain/discomfort, and anxiety/depression [7,20]. Each of these dimensions has three response options, "no problems", "moderate problems", and "extreme problems". Therefore, it classifies a respondent's health status into one of 243 (35) health states. Each health state can be assigned a weighted utility score based on different scoring systems. Due to the unavailability of Moroccan's own value set, we adopted an unweighted scoring rule based solely on answers provided by subjects to the descriptive system. The values are ranging from -0.59 to a maximum of 1 [21]. The second part of the EQ-5D questionnaire is a visual analogue scale (EQ-VAS) [22]. The EQ-VAS is a vertical, graduated (0-100 points) 20 cm 'thermometer', with 100 representing 'best imaginable health state' and 0 representing 'worst imaginable health state. The respondent rated her health on the day of the survey using both the self-classifier system and the VAS.

The Arabic version of the EQ-5D for Morocco was adapted from The United Arab Emirates Arabic version using EuroQol group guidelines and input [7]. An experienced translation consultant who is a native speaker of Moroccan Arabic and fluent in English performed forward translation. Then, a discussion was held with the project manager to produce a first consensus version of EQ-5D for Morocco. A detailed report on the review process outlining the suggested changes was submitted to the EuroQol business management. Emphasis was given to produce a clear and natural-sounding version that is acceptable to respondents in Morocco rather than a direct word by word translation. The first Moroccan consensus version of the EQ-5D was tested on five respondents irrelevant to healthcare professions. Then, a report on respondent testing was sent to the EuroQol Group business management and the Arabic version of the EQ-5D for Morocco was finalized. The translation process has taken into account the socio-cultural context and Moroccan day-life. "Usual activities" was changed to "daily activities". "Prayer" was added to the leisure dimension, "walking" was added to the mobility dimension, and "discomfort" was changed to "not to feel good physically". The Arabic version for Morocco of the EQ-5D questionnaire (Additional file 1) used was granted by the EQ-5D website (http://www.euroqol.org).

### SF-36 health survey

The SF-36 is a multipurpose survey of general health status consisting of 36 items that measure eight scales or health concepts: physical functioning, role physical, bodily pain, general health, vitality, social functioning, role emotional and mental health. Each scale is scored from 0 to 100 with a higher score reflecting a better quality of life [23]. The SF-36 was used as a reference instrument since its psychometric properties have been tested; and it was found both valid and reliable in the ICU setting [1,24]. The Arabic version of the SF-36 has been already validated in Moroccan medical ICU population [16].

### Measurement properties

The acceptability of the EQ-5D was tested by the proportion of missing or invalid responses. Responses to items of the EQ-5D self-classifier were considered invalid if more than one response level was ticked or if a tick was made in between two response levels. Responses to the EQ-VAS were considered invalid when marking was ambiguous (e.g. more than one score marked).

The test-retest reliability refers to the stability of a score derived from serial administration of a measure by the same investigator. It was determined by interviewing 83 survivors, randomly selected, on two occasions separated by 7.5 ± 1 day. The interval of a week was chosen in order to maintain health status between the two administrations. The level of agreement between responses to EQ-5D self-classifier, EQ-5D index and EQ-VAS score of the first and second interview was analysed.

Criterion validity was assessed by comparing responses to the EQ-5D with comparable subscale scores of the SF-36 obtained at the same time. This type of criterion validity is called concurrent validity [25]. The SF-36 was used as a criterion because, compared to the EQ-5D, it is a much more detailed generic measure, which has been shown to cover similar areas of HRQOL [26] and which has been successfully tested and widely used in ICUs [1,16,24].

Construct validity is defined as the ability of the EQ-5D to distinguish between groups that theoretically it should distinguish between. The construct validity was assessed using known-groups comparison to test for hypothesized differences with regard to sociodemographic, clinical, or ICU stay variables.

### Statistical analysis

Descriptive statistics were used to characterize the study sample and their HRQL. Test-retest reliability was assessed using an Intraclass Correlation Coefficient (ICC) for EQ-VAS and EQ-5D index. An ICC above 0.70 is considered to be acceptable [27]. Additionally, the degree of agreement for the EQ-5D self-classifier was evaluated by kappa statistics, which greater than 0.75 indicates excellent agreement, below 0.40 poor, and between 0.40 and 0.75 fair-to-good [28]. For criterion validity with respect to the items of the EQ-5D selfclassifier, we analysed whether the response levels of each item were associated with different comparable SF-36 scores using *t *test. For the EQ-VAS score and EQ-5D index, Pearson and Spearman correlation coefficients with the SF- 36 subscales were respectively calculated. Factors influencing HRQL were studied in univariate analysis using simple linear regression model. Variables with *p *< 0.20 were then included in a multiple linear regression model. The level of significance was 0.05. All statistical analyses were carried out using SPSS version 13.0 (SPSS; Chicago, IL, USA).

## Results

**Figure 1:**
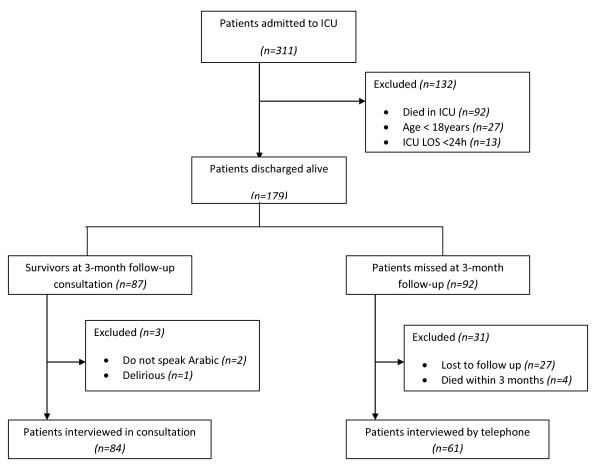
**Patients included and excluded from the study**.

During the study period a total of 311 patients were admitted to the ICU, and 132 were excluded because of death in ICU (n = 92), discharge within 24 h of admission (n = 13), or age under 18 years (n = 27). The remaining 179 patients were discharge alive from ICU. Four patients died before the 3-month follow up, and 27 were lost to follow up. At follow up, 3 survivors were excluded (delirious or unable to understand Arabic), 84 (58%) were interviewed in consultation and 61 (42%) were interviewed by telephone. Thus, 145 survivors completed the EQ-5D (Figure 1). The sociodemographic and clinical characteristics of the study patients (n = 145) are shown in Table 1.

**Table 1:** Sociodemographic and clinical characteristics of study patients (n = 145)

Characteristics	
**Age**, years*; mean ± SD*	38.2 ± 17
**Gender **male*; n(%)*	79 (54)
**Marital status***; n(%)*	
Single	75 (52)
Married	70 (48)
**Minimum educational level***; n(%)*	
None	54 (37)
Primary	41 (27)
Secondary/higher	50 (36)
**ICU length of stay**, days*; mediane [IQR]*	6 [3-10]
**CCI***, n (%)*	
0	92 (64)
1	37 (25)
≥2	16 (11)
**APACHE II**, first 24 hrs; *mean ± SD*	14.1 ± 6
**TISS**, first 24 hrs; *mean ± SD*	20 ± 6
**Mechanical ventilation***; n(%)*	35 (22)
**ICU admitting diagnostic categories***; n(%)*	
Sepsis	36 (24)
Intoxication	33 (23)
Neurologic	23 (16)
Respiratory	21 (15)
Metabolic	14 (9)
Cardiovascular	11 (8)
Miscellaneous	7 (5)

ICU, intensive care unit; CCI, Charlson Comorbidity Index; APACHE II, Acute Physiology and Chronic Health Evaluation II; TISS, Therapeutic Intervention Scoring System

In the total sample, responses to the EQ-5D self classifier are summarized in Table 2. The median of EQ-5D index was 0.52 [0.20-1]. Mean EQ-VAS was 62 ± 20 and ranged from 20 to 100. There were no missing data or invalid responses in the EQ-5D self classifier and EQ-VAS.

**Table 2:** Score distributions of the EQ-5D self classifier in ICU patients

EQ-5D dimensions	**Responses; ***n(%)*		
	
	No problems	Moderate problems	Extreme problems
**Mobility**	80 (55)	46 (32)	19 (13)
**Self-care**	115 (79)	20 (14)	10 (7)
**Usual activities**	77 (53)	40 (28)	28 (19)
**Pain/discomfort**	74 (51)	43 (30)	28 (19)
**Anxiety/depression**	74 (51)	48 (33)	23 (16)

Concerning the results of test-retest reliability in the 83 survivors, the ICCs of the EQ-5D index and EQ-VAS at 7.5 ± 1 day interval were 0.95 and 0.92 respectively exceeding then 0.70. For EQ-5D self classifier, agreement by kappa was excellent in pain/discomfort dimension and fair-to-good in the other dimensions (Table 3).

**Table 3:** Test-retest reliability of EQ-5D

	Kappa-statistic	ICC
**EQ-5D self classifier**		
Mobility	0.73	
Self-care	0.49	
Usual activities	0.66	
Pain/discomfort	0.92	
Anxiety/depression	0.72	
**EQ-5D index**		0.95
**EQ-VAS**		0.92

ICC, Intraclass correlation coefficient; VAS, visual analogue scale

ICC > 0.70 is considered acceptable. Kappa statistics > 0.75 indicates excellent agreement, < 0.40 poor, and between 0.40 and 0.75 fair-to-good

Concerning criterion validity, Table 4 shows the mean scores of SF-36 subscales according to their level of response to comparable EQ-5D items. For all EQ-5D items, mean SF-36 scores were ordered appropriately and were significantly different between the groups; as patients reporting moderate or extreme problems for EQ-5D dimensions at follow up generally had lower mean SF-36 scores than those without such problems. Significant correlations were also noted between EQ5D-index, EQ-VAS and SF-36 subscales (Table 4).

**Table 4:** Criterion validity of EQ-5D with regard to SF-36 subscales

	SF-36 subscales							
	
	GH	PF	RP	RE	SF	BP	VT	MH
**Mobility**								
No problems	71 ± 15	96 ± 6	100 ± 0	80 ± 40	88 ± 22	89 ± 17	64 ± 9	74 ± 14
Moderate/extreme	46 ± 15	54 ± 27	38 ± 48	44 ± 50	47 ± 32	50 ± 32	48 ± 14	66 ± 17
*P*	< 0.001	< 0.001	< 0.001	< 0.001	< 0.001	< 0.001	< 0.001	0.011
**Self-care**								
No problems	63 ± 18	85 ± 18	80 ± 39	72 ± 44	79 ± 25	76 ± 28	60 ± 13	72 ± 15
Moderate/extreme	41 ± 14	37 ± 31	23 ± 42	23 ± 42	23 ± 27	44 ± 13	43 ± 13	64 ± 21
*P*	< 0.001	< 0.001	< 0.001	< 0.001	< 0.001	< 0.001	< 0.001	0.031
**Usual activities**								
No problems	72 ± 12	95 ± 6	96 ± 17	84 ± 36	92 ± 14	86 ± 22	65 ± 8	76 ± 11
Moderate/extreme	42 ± 12	52 ± 28	36 ± 48	36 ± 48	39 ± 28	50 ± 32	45 ± 12	63 ± 18
*P*	< 0.001	< 0.001	< 0.001	< 0.001	< 0.001	< 0.001	< 0.001	< 0.001
**Pain/discomfort**								
No problems	68 ± 17	88 ± 20	91 ± 27	75 ± 43	83 ± 30	96 ± 8	62 ± 13	73 ± 17
Moderate/extreme	48 ± 17	61 ± 30	45 ± 50	48 ± 50	51 ± 31	42 ± 23	50 ± 14	67 ± 16
*P*	< 0.001	< 0.001	< 0.001	0.001	< 0.001	< 0.001	< 0.001	0.038
**Anxiety/depression**								
No problems	68 ± 14	83 ± 24	82 ± 38	92 ± 27	86 ± 23	83 ± 25	64 ± 9	82 ± 8
Moderate/extreme	48 ± 19	66 ± 31	53 ± 50	30 ± 46	47 ± 33	54 ± 32	47 ± 13	57 ± 13
*P*	< 0.001	0.001	< 0.001	< 0.001	< 0.001	< 0.001	< 0.001	< 0.001
**EQ-5D index**	0.79*	0.82*	0.68*	0.64*	0.87*	0.75*	0.76*	0.53*
**EQ-VAS**	0.62†	0.53†	0.48†	0.62†	0.70†	0.47†	0.65†	0.42†

* Spearman correlation coefficients (*p *< 0.001)

† Pearson correlation coefficients (*p *< 0.001)

SF-36, Short Form 36; GH, General Health; PF, Physical Functioning; RP, Role Physical; RE, Role Emotional; SF, social Functioning; BP, bodily pain; VT, vitality; MH, Mental Health

Construct validity of the EQ-5D was confirmed since differences in EQ-5D index and EQ-VAS scores were found between groups of patients (Table 5). Concerning EQ-5D index, in univariate analysis, factors associated with poorer HRQL at follow up were elder age (β = -0.01; *p *< 0.001), married status (β = -0.3; *p *< 0.001), higher educational level (β = -0.2; *p *= 0.010), and longer ICU length of stay (β = -0.01; *p *= 0.049). In multivariate analysis, longer ICU length of stay and higher educational level were both associated with poorer HRQL (β = -0.01; *p *= 0.017 and β = -0.2; *p *= 0.001 respectively). Concerning EQ-VAS, in univariate analysis, factors associated with poorer HRQL at follow up were elder age (β = -0.4; *p *= 0.001) and married status (β = -13.3; *p *< 0.001). In multivariate analysis, men were associated with better HRQL (β = 6.5; *p *= 0.048).

**Table 5:** Construct validity of EQ-5D

	EQ-VAS				EQ-5D index			
	
	Univariate analysis		Multivariate analysis		Univariate analysis		Multivariate analysis	
	
	ß(SE)	*p*	ß(SE)	*p*	ß(SE)	*p*	ß(SE)	*p*
**Age**	-0.4(0.1)	0.001	-0.06(0.1)	0.714	-0.01(0.003)	< 0.001	-0.004(0.003)	0.286
**Gender**								
Female	0		0		0			
Male	5.6(3.4)	0.106	6.5(3.5**)**	**0.048**	0.02(0.1)	0.777		
**Marital status**								
Single	0		0		0		0	
Married	-13.3(3.2)	< 0.001	-8.7(5.1)	0.090	-0.3(0.08)	< 0.001	-0.1(0.1)	0.319
**Educational level**								
None	0		0		0		0	
Primary	-3.9(4.5)	0.384	-6.8(4.5)	0.133	-0.03(0.1)	0.370	-0.02(0.09)	0.78
Secondary/higher	-7.5(4.3)	0.082	-2.6(4.3)	0.535	-0.2(0.1)	0.010	-0.2(0.08)	**0.001**
**CCI**								
0	0				0		0	
≥1	-3.8(4.5)	0.396			-0.1(0.1)	0.164	0.04(0.09)	0.665
**Mechanical ventilation**								
No	0				0			
Yes	2.6(4.0)	0.512			0.00(0.1)	0.997		
**APACHE II**	-0.01(0.3)	0.971			-0.006(0.01)	0.570		
**TISS**	-0.3(0.3)	0.214			-0.01(0.008)	0.122	-0.01(0.01)	0.085
**Length of stay**	-0.02(0.2)	0.897			-0.01(0.006)	0.049	-0.01(0.005)	**0.017**

VAS, visual analogue scale; SE, standard error; CCI, Charlson Comorbidity Index; APACHE II, Acute Physiology and Chronic Health Evaluation II; TISS, Therapeutic Intervention Scoring System

## Discussion

This paper reports the results of the first North-African prospective study concerning the HRQL of ICU patients using the EQ-5D Arabic version. This study provided evidence that the Arabic version of the EQ-5D for Morocco has a good acceptability reliability and validity. Thus, it can be considered as a valid measure of Moroccan patients' health status.

Previous studies have found that the HRQL of patients surviving ICU tends to be significantly poorer than that of samples of the general population [29,30]. Our results showed a relatively high level of HRQL among survivors 3 months after ICU discharge (mean EQ-VAS 62 ± 20 and median EQ-5D index 0.52 [0.20-1]). Other reports using different tools agree with these results [10,16,31]. In our study, moderate to extreme problems in pain/discomfort were reported by 49% of the responders, 47% and 45% of patients reported moderate to extreme problems with usual activities and mobility respectively. But only 21% reported the same level of problems in self-care. Elsewhere, anxiety/depression was the only dimension causing moderate to extreme problems in more than 50% of the patients. These findings are similar to that of Granja et al. [10] using the EQ-5D 6 months after ICU discharge where pain/discomfort and anxiety/depression were the most frequently reported problems. Other studies using different tools [29,31] reported also the same findings in ICUs where emotional problems and anxiety/depression were the most frequent one. Concerning EQ-5D index, in order to eliminate the possible influence that social preferences may have on the score, an unweighted scoring rule was developed based solely on answers provided by subjects to the descriptive system [21]. This unweighted scoring strategy was used in our study because it simply combines the answers provided by the subjects to each of item of the questionnaire.

The EQ-5D was well accepted and understood by ICU patients at follow-up, as shown by the lack of missing and invalid responses. Furthermore, EQ-5D is known to be a simple and short questionnaire easily understood and answered by the patient; these can make it an instrument of choice in ICU patients [10].

Because comparative studies require instruments in several languages, cross-cultural adaptation of HRQL questionnaires should be entertained carefully [32]. Although EQ-5D has been translated into more than 50 different languages including classic Arabic, few publications have described the process of cross-cultural adaptation and validation in detail and none is available in the North-African countries [15]. Before being used in a socio-cultural and specific setting, the EQ-5D needs to demonstrate satisfactory psychometric properties because of its brevity and because of information for each dimension being derived from only one item [13]. The EQ-5D demonstrated good test-retest reliability. The ICC was excellent in our study (0.95 and 0.92 for the EQ-5D index and EQ-VAS respectively). Kappa-statistics were satisfactory in overall the five EQ-5D dimensions (0.66-0.92). Several other validated EQ-5D versions from foreign countries have also demonstrated good reliability with comparable results [11,12,14].

The SF-36 is one of the most widely used generic health status measure that can be considered as a "Gold Standard" in ICU settings [1,24]. Its psychometric properties have already been proved in a Moroccan medical ICU [16]. Comparison of EQ-5D response with subscale scores of SF-36 showed excellent criterion validity, the EQ-5D index and EQ-VAS correlated well with the eight SF-36 health domains. So was found in a German study comparing EQ-5D and SF-36 in cardiac rehabilitation [11] and another one in patients with inflammatory bowel disease [33].

The HRQL of ICU patients has been investigated at various follow-up times. Assessment of HRQL at 90 days may be ideal for the detection of ICU related sequelae, because more time passes the larger are the effects of increasing age and possibly new comorbidities on HRQL [34]. The construct validity of the EQ-5D was confirmed in our study. In multivariate analysis, linear regression model indicated that women had poorer HRQL (EQ-VAS), a fact that has been already reported in the literature where women report worse health than men in ICUs [16]. Surprisingly, we found that ICU patients with high education levels had poorer HRQL contrary to what has been reported in the literature [10]. The differences between these findings and ours can be explained by cultural specificities, concerning values, perceptions, and expectations regarding health care [35]. Patients with a high level of culture and education may be more emotionally and/or physically demanding; while those with low education levels because the lack of culture, their only religious beliefs make them consider their health status as God's will and may express then better HRQL. Moreover, poorer HRQL related with high education level can be also explained by the fact that questionnaires were self-completed by this category of patients, this may have facilitated the fully expression of health status assessment. ICU patients with less education level may have been intimidated by the presence of the investigator while completing questionnaires. Longer ICU length of stay was also associated with poorer HRQL, a fact that is common in earlier studies [10,16]. In our study, age was not found to be related to HRQL. In fact, the impact of age on HRQL is controversial in ICUs: impairment of HRQL with elderly has been previously reported [10] whereas other studies showed no influence of age [36].

There are some limitations in the study. First, the EQ-5D is a self-administered questionnaire, but the problem of the low literacy in Morocco yielded to a self-administration or administration by the same investigator following the level of education of the patients. This may introduce bias in the results; however no consensus is available concerning the problem of administrating questionnaires in low literacy populations [37]. Second, a large number of the patients were excluded from the study; this may have introduced bias into the results. Third, the EQ-5D was administered 3 months after ICU discharge; HRQL may then vary over time.

## Conclusions

We conclude that the Morrocan Arabic EQ-5D has good acceptability and validity in measuring health status after intensive care in Morocco. Three months after ICU discharge, women and survivors with higher educational level and longer ICU length of stay expressed poorer HRQL. In our context, the use of the EQ-5D can be recommended in order to provide a better picture of the HRQL among ICU patients. These data provide a basis for further studies of the EQ-5D in Morocco.

## Abbreviations

APACHE II: Acute Physiology and Chronic Health Evaluation II; CCI: Charlson comorbidity index; EQ-5D: EuroQol-5-Dimension; HRQL: Health-related quality of life; ICC: Intraclass correlation coefficient; ICU: Intensive care unit; SF-36: Short form 36; TISS: Therapeutic intervention scoring system; VAS:Visual analogue scale.

## Competing interests

The authors declare that they have no competing interests.

## Authors' contributions

IK drafted the manuscript, participated in the design of the study, performed the statistical analysis and interpretation of data. JB participated in the acquisition of data. TD participated in the acquisition of data. KA assisted in the interpretation of the results. NM assisted in the interpretation of the results. AZ helped to prepare the manuscript. AAZ helped to prepare the manuscript. RA conceived of the study, participated in the design of the study, performed the statistical analysis and interpretation of data, and gave the final approval of the manuscript. All authors read and approved the final manuscript.

## Supplementary Material

Additional file 1 **The Arabic version for Morocco of the EuroQol-5Dimension questionnaire**. Click here for file
