# Supplementary material for: Measuring quality of life after intensive care using the Arabic version for Morocco of the EuroQol 5 Dimensions
Source: BMC Res Notes. 2012 Jan 22;5:56. doi: 10.1186/1756-0500-5-56 (PMC3293002; doi:10.1186/1756-0500-5-56)
Supplement: Additional file 1 — The Arabic version for Morocco of the EuroQol-5Dimension questionnaire. [file 1756-0500-5-56-S1.DOC]

***The Arabic version for Morocco of the EuroQol-5Dimension***

**عافاك دير علامة على مربّع واحْد فْكل مجموعة دٍيال الأجوبة وْ الّلي كايْوافقْ حالتْك الصّحية دْيال اليوم**.

#### الحركة و الْمْشٍي

- ما عْندي حتّى مشاكل فلْمشي ❑
- **عْندي شْويّة دْيال المشاكٍل فلْمشٍي** ❑
- أنا قابْطْ الفْراشْ ❑

**تقْدّ براسِْكْ وْ تقادّ حاْلتكْ**

- نْقدْرنْقابْل راسي بوحْدي بْلا مشاكٍل❑
- عْندي شٍْويّة دْيال المشاكٍل فْلْغْسيل وْ لْبيسْ الحْوايج ❑
- ما كانْقدْرشْ نْغسْلْ وْ نْلْبسْ حْوايجي بوحْدي ❑

الأنشطة اليوْميّة

**( مثلا الخْدْمة, القْراية, شْغالْ الدّار, شْغالْ العائٍلة, شْغالْ الْفراغْ, الصّلاة)**

- ما عْنْدي حْتّى مشاكٍل فلأنْشٍطة دْ يالي اليوْميّة ❑
- عْنْدي شْويّة دْيال المشاكٍل فْلأنْشٍطة دْ يالي اليوْميّة❑
- ما كانْقدْرشْ نْديرْ اْلانْشٍطة دْيالي اليوْميّة❑

**الْحْريقْ/ الرّاحة فالدّات**

- ما فٍيّاشْ الحْريقْ و مْرتاح فْداتي❑
- فٍيّا شْويّة دْيالْ الحْريق و مامْرتاحش فْداتي❑
- فٍيّا بْزّافْ دْيالْ الحْريقْ و مامْرتاحش فْداتي ❑

#### القلق / الاكتٍئاب

- ما مْقّلّقْ ما مْكتائب❑
- مْقّلّقْ وْلا مْكتائبْ شْوٍيّة❑
- مْقلّقْ وْلا مْكتائبْ بْزّافْ ❑

**Visual Analogue Scale**

**90**

**80**

**70**

**60**

**50**

**40**

**30**

**20**

**10**

**100**

**الصّحة الضّعيفة اللّي** **يْمكنْلك تّخايلْها**

**0**

**الصّحة المْزيانة اللّي** **يْمكْنلك تّخايلْها**

باشْ نْعاوْنو النّاس يْبٍيّنولينا مْزيانْ حالْتْهم الصّحيّة, رْسمْنا واحْد الخطّ مْشْرّطْ (بْحال ميزان السّخانة) فٍيه الرّقم 100 هو الصّحة المْزيانة اللّي يْمْكنلْك تّخايْلها وْ 0 هو الصّحة الضّعيفة اللّي يْمْكنلْك تّخايْلها.

بْغينا عافاكْ تْبٍيّنْ لينا فْهاد الخطّ لْمْشرّطْ قْدّاشْ كانْت صْحّتْك مْزْيانة وْلا

ضْعيفة فْهادْ اليوم.

عافاكْ رْسْم خطّ تيْبدا مْن المربّع اللّي الْتحْت وْ تايْمشي حْتّال شي نْقْطة

مْن النّقاطي الموجودين فالْخطّ المْشرّط وْ اللّي كتْبيّن حالتْك الصّحيّة

دْيال اليوم.

حالْتْكْ الصّحيّة

دْيالْ اليومْ
